# Design and evaluation of a group-based integrative expressive art therapy intervention for mental health among pregnant women in China: a pilot pre-post study

**DOI:** 10.3389/fpsyt.2026.1850434

**Published:** 2026-09-03

**Authors:** Xue Feng, Quanlun Li, Ling Huang, Hongting Yan, Chunmei Lan, Yu Ling, Ruimin Ling, Yini Li, Hua Yu

**Affiliations:** 1 School of Information and Management, Guangxi Medical University, Nanning, Guangxi, China; 2 Nanning Mental Health Association, Nanning, Guangxi, China; 3 The Second Affiliated Hospital of Guangxi University of Chinese Medicine, Nanning, Guangxi, China

**Keywords:** anxiety, art therapy, expressive art therapy, pregnancy, willingness-to-pay

## Abstract

**Background:**

Pregnant women in China face various emotional difficulties during pregnancy but lack adequate mental health support. This study aimed to design and evaluate a group-based integrative expressive art therapy intervention for addressing emotional and psychological concerns in this population.

**Methods:**

A quasi-experimental design with pre- and post-intervention assessments were delivered from July to December 2025. We developed a five-session expressive art therapy intervention employing multiple art modalities, including painting, doodling, hand-making, OH cards, music as well as storytelling. In the secondary analyses, we selected a control group (N = 14) to compare participants’ perceptions of art therapy and willingness-to-pay against those in the intervention group (N = 14).

**Results:**

After the intervention, significant reductions in both state and trait anxiety were observed (p<0.05). Qualitative analyses revealed improvements in emotional awareness, self-expression, cognitive restructuring, stress relief, social support, social connection, self-acceptance, and self-efficacy. In the secondary analyses, participants who received intervention reported more positive perceptions of its effects in emotion regulation, anxiety reduction, and mother–child relationship enhancement compared with non-participants (p<0.05), while no significant differences were found in willingness-to-pay (p=0.7).

**Conclusions:**

This pilot study demonstrates the feasibility and effects of a group-based integrative expressive art therapy for pregnant women in China. The findings support the application of expressive art therapy as a mental health service and provide implications for the development of related health policies.

## Background

Over 80% of women during pregnancy experience different levels of emotional difficulties (1); approximately 10-25% of them may suffer from anxiety and depression (2). In particular, prenatal anxiety is psycho-biological process driven, for example, by changing hormonal status (3, 4); it correlates with several concerns, including worries about undesirable fetal and maternal outcomes, embracing the new maternal role, fear of giving birth, and uncertainties about various stages of pregnancy (5). Up to now, plenty of interventions have been implemented to support the mental health of this population. For instance, Mazraei Farahani et al. (2025) applied a solution-focused group counseling intervention that significantly reduced pregnancy anxiety level (6).

In addition to traditional counseling approaches (7), art therapy interventions have also demonstrated efficacy in enhancing psychological well-being among this population. Art therapy leverages creative expression as a vehicle (8), effectively bypassing the stigma of mental health and empowering self-expression across varied cultural contexts. According to a 2023 study by Sharifpour et al, a series of painting-based art therapy interventions performed among Iranian pregnant women showed significant improvement in sleep quality (9). A 2023 meta-analysis of 21 studies involving 2,815 pregnant and postpartum women found that art-based interventions significantly alleviated participants’ anxiety and depression levels (10).

Despite significant unmet mental health needs among Chinese women in the prenatal and postpartum periods, art therapy remains substantially underutilized in clinical settings. Moreover, China faces a severe shortage of psychotherapists, with only approximately 5,000 to 6,000 registered psychotherapists nationwide (11), which indicates an alarming scarcity in the mental health workforce. In fact, psychotherapists or psychotherapy service are still not available in most local hospitals in China, indicating that maternal mental health care is primarily provided by obstetricians, nurses, or other clinicians. Previous literature on Chinese pregnant women has predominantly documented music therapy during childbirth (10). A 2024 study illustrated that integrating music therapy with doula-assisted midwifery care during labor significantly reduced postpartum depression rates and enhanced quality of life for perinatal women (12). However, the depth and variety of art therapy engagement in mental health interventions across pregnancy stages remain notably insufficient.

Within the contemporary Chinese context, expressive art therapy remains a nascent and frequently misinterpreted concept; it is often equated with recreational art-making or perceived as mere “art as therapy”. Pervasive mental-health stigma further suppress public awareness and acceptance of art therapy as a mental health service (13), thereby restricting pregnant women’s access to this intervention.

Despite the challenges mentioned above, integrating a group-based intervention that combines expressive art therapy into existing prenatal education systems is a feasible approach, which may improve pregnant women’s healthcare experience and address the limitations of mental health support in China. Therefore, this study aims to develop a group-based integrative expressive art therapy program for clinical application with pregnant women; evaluate its effects on anxiety, depression, and health-related quality of life; and compare beliefs about expressive art therapy and willingness-to-pay (WTP) between intervention participants and non-participants.

## Methods

### Study design

We applied a quasi-experimental design from July to December 2025 for this preliminary study. Participants in the intervention group completed pre- and post-intervention assessments via online questionnaires, followed by a semi-structured qualitative interview. In addition, we enrolled a control group of pregnant women without any art therapy experience, recruited from a cross-sectional study. Participants in control group were assessed in alignment with the study’s recruitment window and full intervention period. All participants were recruited at the Second Affiliated Hospital of Guangxi University of Chinese Medicine, Nanning, China.

### Sample and setting

We included pregnant women aged ≥18 years who were willing and able to appear at the study site, self-identified emotional concerns requiring professional support, and could complete an online questionnaire. Exclusion criteria were a diagnosed mental disorder (e.g., major depression, bipolar) or expected delivery within one month at the time of survey. Potential participants were recruited via online advertisements. Clinical staff also introduced the intervention to eligible pregnant women during routine clinical consultations. All individuals who expressed interest upon receiving study information underwent eligibility screening. Eligibility was confirmed via a survey platform Wenjuanxing (https://www.wjx.cn). Intervention took place in a private room in the hospital under the guidance of the aboard-certified psychotherapist and counsellor trained in expressive art therapy. We also offered parallel sessions on newborn care for the partners and family members of the pregnant women; these parallel sessions were led by the clinicians. Informed consent were obtained from all the participants, and the study was approved by the Ethics Committee of Guangxi Medical University (approval No.KY20250263).

### Procedures

We developed a series of group-based integrative expressive art therapy sessions, which were a five-week program, two-hour session per week, each centered on a distinct theme (**Table 1**), varying from “mother-child relationship” to “group painting therapy with peer support”. All sessions were led by a trained art psychotherapist. We combined expressive art therapy with drawing, doodling, hand-making (14), OH-card guided narrative therapy, as well as music relaxation exercise across all sessions. During session 4, participants were asked to invite their family members to attend the group sessions. Since most pregnant women arrived at the study site accompanied by their relatives, nurses and other clinicians delivered parallel educational classes on childcare guidance for accompanying family members at every session.

**Table 1:** Themes and objectives of the integrative expressive art therapy intervention among pregnant women (N = 14).

No.	Theme	Objectives
1	Nurturing the mother–child bond	to become familiar with expressive art therapy to strength emotional awareness and explore the mother-infant relationship through self-inquiry.
2	Navigating difficult motions in Pregnancy	to enhance maternal self-efficacy through strengthening self-acceptance and identifying adaptive coping strategies during pregnancy period.
3	Finding calm in pregnancy’s uncertainties	to acquire skills to regulate uncertainty and anxiety, and strengthen maternal confidence in navigating pregnancy-related challenges.
4	A family therapy gathering (family members invited)^*^	to enhance family support and maternal–family interaction by leveraging family resources to foster a positive perception of childbirth.
5	Group painting therapy with peer support	to facilitate participant interaction, strengthen socio-emotional connections, and enhance peer social support among pregnant women.

*participants attended Session 4 were accompanied by their family members.

### Measures

#### Depression state

We employed commonly-used self-reported screening questionnaire-PHQ-9 to assess the presence and severity of depressive symptoms of the participants occurring within two weeks prior to the intervention. PHQ-9 consists of nine items (15–17), such as depressed mood, sleep problems, and fatigue. For example, “feeling down, depressed, or hopeless (in Chinese) were asked. Response items were scored on a scale from 0 (never) to 3 (almost every day), with total scores ranging from 0 to 27. The PHQ-9 (Chinese version) demonstrated a Cronbach’s alpha exceeding 0.8 in Chinese populations (17).

#### Anxiety state

Anxiety was evaluated using the short Chinese version of the State-Trait Anxiety Inventory (STAI); each subscale comprised six items (i.e., STAI-S-6 and STAI-T-6) (18–20). For example, item from the STAI-S-6 reads”I feel upset”(in Chinese), whereas the item from the STAI-T-6 states”I feel pleasant”(in Chinese). The total scores for both STAI-S-6 and STAI-T-6 range from 0 to 24. Higher scores indicate greater levels of state/trait anxiety. A cut-off point of 15.5 for state anxiety and 17.5 for trait anxiety was used to classify low and high levels, respectively. The Cronbach’s alpha efficients were over 0.8 for both STAI-S-6 and STAI-T-6 in Chinese population (20).

### Health related quality of life

We employed the EQ-5D-5L (Chinese version) to assess health related quality of life (21, 22). The instrument, developed by the EuroQol Group, is among the most widely used generic preference-based measures. It quantifies five main dimensions of health-related quality of life, namely mobility, self-care, usual activities, pain/discomfort, and anxiety/depression. For example, participants were asked the item “I have no problems in walking about” (in Chinese). The descriptive system of EQ-5D-5L comprises five response levels for each dimension, specifically, “no problems”, “slight problems”, “moderate problems”, “severe problems”, and “extreme problems”. The EQ-5D-5L also includes a visual analog scale (EQ VAS), which ranges from the best imaginable health state (100) and the worst imaginable health state (0). The EQ-5D-5L defines 3125 (55) health states. We derived the EQ-5D-5L index scores by mapping responses onto the China-specific EQ-5D value sets (17). EQ-5D-5L has been demonstrated with good psychometric properties in Chinese populations, including pregnant women (23, 24).

### Belief on expressive art therapy

We used the Health Belief Model (HBM) (25) as a theoretical framework to guide the development of questions assessing beliefs about expressive art therapy. The HBM is a widely used framework for understanding health behaviors. The HBM constructs in this study included: perceived severity, perceived susceptibility, perceived barriers, perceived benefits, cues to action, and self-efficacy. Participants rated their agreement with statements for each construct on a four-point Likert scale, ranging from 1 (strongly disagree) to 4 (strongly agree).

### Willingness-to-pay

Willingness-to-pay was elicited via the contingent valuation method (CVM), which is validated stated-preference approach to monetize non-market health benefits through discrete-choice experiments. In this study, respondents were presented with a dichotomous choice to indicate whether they were willing to pay for health gains at the provided amount. Based on the initial response, three additional amounts were presented with corresponding choices. Specifically, participants were asked the following statement

“Globally, 10–20% of women during pregnancy experience depression, anxiety or other psychological distress each year. These conditions not only affect the mother’s mental and physical health but may also impede fetal development. To address this challenge, we have introduced an expressive art therapy program tailored for expectant mothers. The course uses creative art-making activities, such as drawing OH-card, and story telling, to help expectant mothers build an emotional bond with their unborn baby, reduce anxiety and facilitate communication with family members.”

### Other independent variables

The survey collected socio-demographic data, including gender (male, female), age group (18-24, 25-30, 31-40, >40 years), place of residence (rural, urban), annual household income level (<20k RMB, 20k-100k RMB, 100k-200k RMB, >200k RMB), highest education level (high school/diploma or below, Bachelor’s degree, Master’s degree and above), occupation, trimester (weeks)/postpartum period, and whether this was the first pregnancy.

### Statistical analyses

Statistical analyses were conducted using R version 4.1.2. (R Foundation for Statistical Computing, Vienna, Austria). Descriptive statistics were used to characterize the sample; continuous variables were summarized using means and standard deviations, while categorical variables were expressed as frequencies and percentages. Paired t-tests were used to examine pre-post differences in outcomes following the intervention. State anxiety, measured by the STAI-S, was assessed at baseline, after the first intervention session, and upon completion of all sessions. All other scales, including the PHQ-9, were administered at both baseline and the final session. In the secondary analyses, to compare the difference between intervention and control group, we employed propensity score matching (PSM) to address baseline disparities. The initial sample prior to propensity score matching included 92 participants recruited within the identical enrollment and intervention time frame; after 1:1 nearest-neighbor matching, the final matched sample was reduced to 14 participants. Variables used in the PSM included age group, place of residence, education level, and annual household income level. P values were calculated using independent two-sample t-tests for the continuous variables; the Mann-Whitney U test was conducted to compare willingness-to-pay between the intervention and control groups. We calculated differences in total scores for the PHQ-9, STAI-S, STAI-T, EQ VAS, and utilities derived from the EQ-5D-5L.

### Qualitative analyses

Follow-up semi-structured interviews were conducted with 10 participants (6 in-person, 4 by telephone) after all the interventions. Two participants who attended only the initial session submitted written feedback. The data were analyzed using inductive thematic analysis (26). Recorded interviews were transcribed via built-in software; each lasted 10 to 15 min and was delivered by trained research staff. The inductive coding process comprised an initial round of open-coding followed by a second round of axial coding. These were ultimately formulated into seven core themes, including 1) emotion awareness, 2) self-expression, 3) cognitive reconstruction, 4) stress reduction, 5) social support, 6) social connection, and 7) self-acceptance& self-efficacy.

## Results

A total of 14 pregnant women who met the inclusion criteria were enrolled in the intervention group; an additional 14 participants were selected in the control group based on PSM. More than 50% were aged 25–30 and all held a bachelor’s degree or higher (**Table 2**). Approximately 80% lived in urban areas, and about 50% reported household income of 20k-100k RMB. More than 60% of pregnant women were experiencing their first pregnancy; most were in the second or third trimester.

**Table 2:** Descriptive analyses (N = 28).

Variables	Total		Intervention group		Control group	
	N	%	N	%	N	%
Female	28	100%	14	100%	14	100%
Age						
20-24	2	7.14%	0	0.0%	2	14.4%
25-30	15	53.57%	9	64.3%	6	42.8%
31-40	11	39.29%	5	35.7%	6	42.8%
Education level						
Bachelor	26	92.86%	13	93.0%	13	93.0%
Master and above	2	7.14%	1	7.0%	1	7.0%
Place of enrollment						
Urban	22	78.57%	11	78.6%	11	78.60%
Rural	6	21.43%	3	21.4%	3	21.40%
Occupation						
Employee	10	35.71%	2	14.29%	8	57.14%
Homemaker	7	25.00%	4	28.57%	3	21.43%
Teacher	6	21.43%	4	28.57%	2	14.29%
Free-lancer	5	17.86%	4	28.57%	1	7.14%
Family income						
20k RMB below	4	14.29%	3	21.43%	1	7.14%
20k-100k RMB	14	50.00%	6	42.86%	8	57.14%
100-200k RMB	5	17.86%	2	14.29%	3	21.43%
200k+ RMB	5	17.86%	3	21.42%	2	14.29%
Trimester (weeks)/postpartum period						
0–12 weeks	2	7.14%	2	13.3%	0	0.00%
13–28 weeks	11	39.29%	8	53.3%	3	21.43%
29–40 weeks	13	46.43%	4	26.7%	9	64.29%
after childbirth	2	7.14%	0	0.00%	2	14.29%
First pregnancy*						
Yes	18	69.23%	9	69.20%	9	75.00%
No	8	30.77%	5	30.80%	3	25.00%

*The two missing data points in the control group resulted from two participants being in the postpartum period.

At baseline, the mean PHQ-9 score was 6.07, indicating mild depressive symptoms. The mean STAI scores for both state and trait anxiety were approximately 13, reflecting mild anxiety levels among the pregnant women in the intervention group. In this pre-post intervention study (N = 14), significant reductions (~16%) were observed in both state anxiety (13.07 to 10.93, p=0.047, Hedges’g [95%C]=0.56[0.01, 1.13]) and trait anxiety (12.79 to 10.62, p=0.03, Hedges’g [95%C]=0.65[0.05, 1.22]) (**Table 3**). Variations in scores on the PHQ-9, utility, and EQ-VAS remained non-significant; the modest observed improvements were characterized by considerable variability, suggesting uncertainty attributable to confounding factors during the intervention period.

**Table 3:** Difference on depression states, anxiety states, and health related quality of life before and after the intervention.

Variables	Pre	Post	p value^a^	Hedges’g [95%C]
	mean ± SD	mean ± SD		
N	14	14		
STAI-S	13.07 ± 3.01	10.93 ± 3.29	0.047	0.56[0.01, 1.13]
N	12	12		
STAI-S	11.92 ± 3.01	10.83 ± 3.30	0.097	0.51[-0.09, 1.08]
STAI-T	12.79 ± 3.53	10.62 ± 2.88	0.03	0.65[0.05, 1.22]
PHQ-9	5.50 ± 4.09	5.58 ± 4.74	0.95	-0.02[-0.58, 0.57]
utility	0.80 ± 0.23	0.84 ± 0.16	0.45	-0.21[-0.74, 0.33]
VAS	72.08 ± 25.54	79.62 ± 20.82	0.18	-0.39[-0.93, 0.17]

^a^P values are presented based on paired t-tests. SD, standard deviation; PHQ-9, Patient Health Questionnaire-9; STAI-S, the State-Trait Anxiety inventory- state sub-scale; STAI-T, the State-Trait Anxiety inventory- trait sub-scale; VAS, visual analog scale.

In the secondary analyses, severe imbalance in age and education was resolved after PSM, with education and residence perfectly balanced and mild, acceptable residual imbalance in age and income. **Table 4** summarizes the results derived from these analyses; the two groups showed no statistically significant differences in depression, state anxiety, trait anxiety, overall health status, and health-related quality of life (all p > 0.05), indicating good homogeneity between groups. Regarding expressive art therapy-related beliefs, no differences were observed in expressive art therapy knowledge (Q1) or the severity of self-perceived mental health states (Q2) (both p > 0.05). However, intervention group reported significantly higher perceived harm from mental health illness than the control group (p = 0.003). Regarding perceived benefits of expressive art therapy, the intervention group exhibited significantly stronger perceptions on expressive art therapy in reducing anxiety (Q5, p = 0.04), improving overall health (Q6, p = 0.01), enhancing emotion regulation (Q7, p = 0.008), and promoting mother-child relationships (Q9, p = 0.01) compared to the control group; the difference in perceived benefit for self-expression (Q8, P = 0.053) was marginally significant. For cues to act (clinician recommendation Q10 and persistence Q11), no statistically significant differences were observed between the two groups (p > 0.05). A Mann-Whitney U test was performed to compare willingness-to-pay between groups. The analysis revealed no statistically significant difference (U = 91.0,Z = -0.380, p = 0.769).

**Table 4:** Differences in perceptions of expressive art therapy between intervention (N = 14) and control (N = 14) groups.

Variables	Intervention	Control	p value^a^	Hedges’g^b^[95%C]
	mean ± SD	mean ± SD		
Baseline PHQ-9	6.07 ± 4.09	5.00 ± 2.99	0.44	0.29[-0.44,1.01]
Baseline STAI-S	13.07 ± 3.01	11.5 ± 3.82	0.24	0.44[-0.29,1.17]
Baseline STAI-T	12.79 ± 3.53	11.36 ± 3.41	0.29	0.40[-0.33,1.12]
Baseline utility	0.78 ± 0.21	0.87 ± 0.10	0.16	-0.53[-1.25,0.21]
Baseline VAS	71.00 ± 25.54	80.43 ± 15.34	0.25	-0.44[-1.16,0.30]
Belief about expressive art therapy				
Q1 Art therapy knowledge				
	2.86 ± 0.77	3.07 ± 0.92	0.51	-0.25[-0.97, 0.48]
Q2 Perceived current mental health state (severity)				
	3.43 ± 1.09	4.00 ± 0.96	0.15	-0.54[-1.27, 0.20]
Q3 Perceived harm of mental health illness				
	1.93 ± 0.92	3.36 ± 1.34	0.003	-1.21[-1.99,-0.41]
Perceived benefit of expressive art therapy				
Q4 Perceived effectiveness on mental health issues from expressive art therapy				
	2.00 ± 0.56	2.50 ± 0.94	0.1	-0.63[-1.36, 0.12]
Q5 Perceived benefit of expressive art therapy on anxiety reduction				
	1.93 ± 0.48	2.50 ± 0.86	0.04	-0.80[-1.55,-0.04]
Q6 Perceived benefit on positive affects on overall health from expressive art therapy				
	1.79 ± 0.43	2.36 ± 0.63	0.01	-1.03[-1.79,-0.25]
Q7 Perceived benefit on emotion regulation from expressive art therapy				
	1.86 ± 0.54	2.43 ± 0.51	0.008	-1.06[-1.82,-0.28]
Q8 Perceived benefit on self-expression from art therapy				
	1.93 ± 0.48	2.36 ± 0.63	0.053	-0.74[-1.48, 0.01]
Q9 Perceived benefit on mother-child relationship from expressive art therapy				
	1.93 ± 0.48	2.43 ± 0.51	0.01	-0.98[-1.74,-0.21]
Q10 Cue to act for expressive art therapy from clinician recommendation				
	2.07 ± 0.62	2.36 ± 0.84	0.32	-0.38[-1.10, 0.35]
Q11 Cue to act for expressive art therapy -persistence				
	2.07 ± 0.62	2.64 ± 0.93	0.07	-0.70[-1.44, 0.05]
Willingness-to-pay	8 (57.1%)	9 (64.3%)	0.77^§^	/
Highest price to pay for 5 sessions (RMB)	235.71 ± 313.43	134.29 ± 128.29	0.28	-0.14[-0.86, 0.58]

a P values are presented based on independent two-sample t-tests. ^b^Hedges’ correction for small-sample bias.^§^Mann-Whitney U test was perform for willingness-to-pay. RMB, Renminbi (Chinese official currency); SD, standard deviation; PHQ-9, Patient Health Questionnaire-9; STAI-S, the State-Trait Anxiety inventory- state sub-scale; STAI-T, the State-Trait Anxiety inventory- trait sub-scale; VAS, visual analog scale.

Among participants unwilling to pay for this expressive art therapy, affordability was the primary concern (40%), followed by lower prioritization of mental health and preference for alternative services. On the other hand, those who were willing to pay emphasized prior class feedback and location convenience as key decision-making factors. Overall, 64% of participants reported increased willingness to pay for this group-based integrative expressive art therapy if covered by health insurance (data not presented).

### Qualitative analyses

Seven major themes were generated based on the semi-structured interviews with 12 participants who agreed to on-site or telephone interviews, including 1) emotion awareness, 2) self-expression, 3) cognitive restructuring, 4) stress reduction, 5) social support, 6) social connection, and 7) self -acceptance & self-efficacy.

### Theme 1. Emotion awareness

Mother 5:”While I painted, I tried to show how I felt at that moment. When I shared my pain to the group … saying it out loud helped me notice my own feelings.”

The drawing, doodling, OH cards, narrative therapy, and other activities provided pregnant women with opportunities to confront their inner feelings directly. Through creative expression and group interaction, they gradually became aware of emotions such as unhappiness during pregnancy. With guidance from instructors and through self-reflection, they gained clearer insights into the specific situations and underlying causes that triggered these emotions, thereby significantly enhancing their emotional awareness.

### Theme 2. Self-expression

Mother 1:”Under instructor’s guidance … I could just paint whatever I wanted without caring how it looked … every little bit meant something to me.”

The intervention fostered an inclusive atmosphere, allowing pregnant women to express their emotions freely, through drawing, coloring, verbal sharing, and other creative modalities without concern for artistic skill or expressive ability. This low-pressure ways of expression enabled them to share their thoughts effectively, and enhanced self-expression.

### Theme 3. Cognitive restructuring

Mother 6: “I used to think only about me. Now I ask, ‘What can I do to help?’…I talk with my family more … so both sides feel better.”

Through sharing, interactive discussions, doodling exercises, and supportive guidance from instructors, participants learned to view conflicts with family members from new perspectives; participants exhibited a shift away from self-centered thinking, and adjusted their communication patterns with their family members. This process fostered meaningful cognitive restructuring, which in turn contributed to improved psychological well-being.

**Figure 1:**
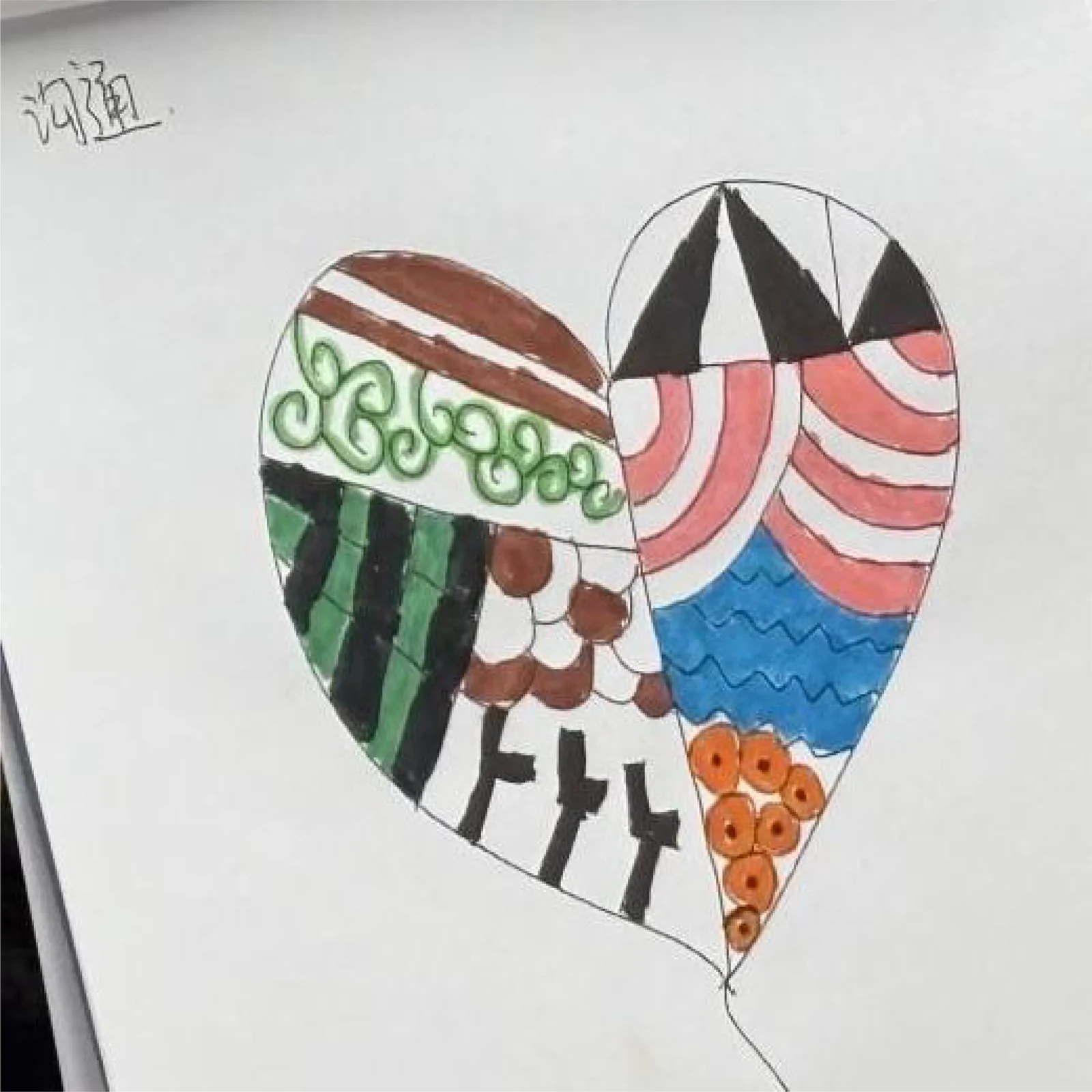
Doodling.

**Figure 2:**
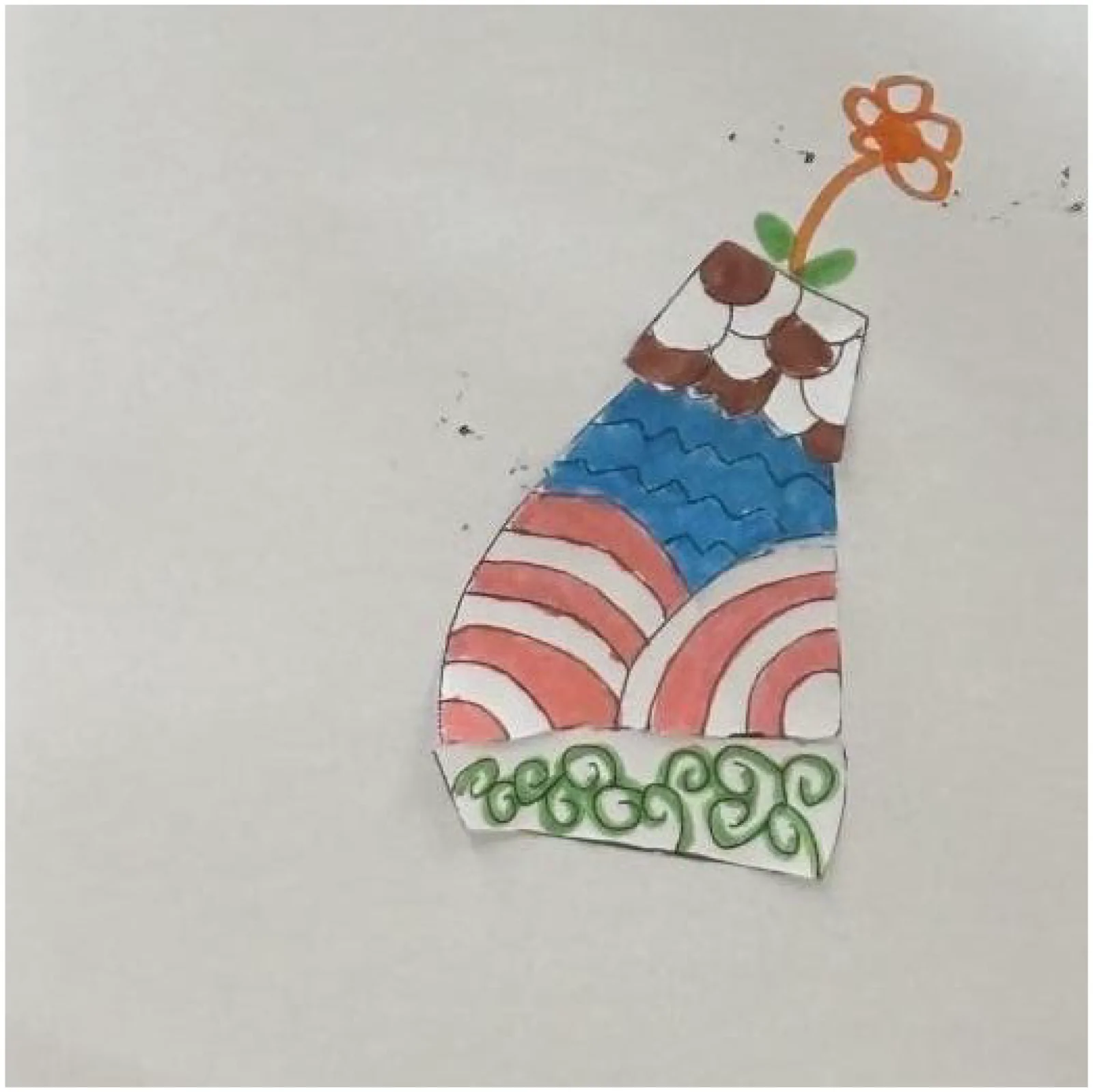
After reconstructing.

Participant mother 7 created a heart-shape painting (**Figure 1**): its left half, in darker shades, represented her extended family (parents, in-laws and elders), visualizing generational value conflicts; the other bright half symbolized her new family (with her husband), highlighting her new-life aspirations and the inter-generational communication problems. During the reconstruction stage (**Figure 2**), she merged the two color zones into a single vase shape. The vase retained the bright tones of her new family, while the once conflicting dark fragments from the heart’s other half were redefined as essential supportive elements. A small flower blooming from the vase symbolized the renewed inter-generational dialogue that fostered her personal growth, marking her transition from relational disconnection to recovery. Additionally, this participant shared positive feedback on her family communication improvements in the subsequent session, offering valuable insights to other participants with similar concerns.

### Theme 4. Stress reduction

Mother 1: “After all these classes, I feel way calmer.”

Mother 6:”Even if the problem’s still there, just talking about it make me feel better.”

The intervention effectively relieves stress by creating a safe, supportive space: 1) weekly sessions for sharing one’s current state and personal situations help mothers release emotional tension and ease psychological burdens; 2) peer experience-sharing helps mothers recognize that such stress is common, motivating them to adopt a more proactive approach to stress management; and 3) the expressive art therapy session complements verbal expression, providing an alternative medium for releasing emotions.

### Theme 5. Social support

Mother 11: “I was in the hospital that week and felt terrible. I came to class right from the ward. Everyone cheered me on, and it really moved me. After I shared my story, I felt so much better.”

Mother 5: “…with the instructor’s guidance and my fellow participants’ sharing, I realized that everyone is going through similar challenges … it made me feel that I need to learn how to handle this stress myself.”

The intervention effectively delivered social support through three interconnected dimensions—emotional, informational, and belonging-related support—which collectively enhanced participants’ psychological well-being. For example, pregnant women felt seen and comforted through peer encouragement and empathetic listening; as participant mother 11 described, she felt “much more at ease” after one session. Additionally, they gained practical coping strategies by hearing others’ experiences. As one mother noted, “everyone is going through similar challenges,” which reflected a sense of belonging and mutual validation. Together, these interconnected dimensions of support empowered pregnant women to navigate emotional difficulties with greater resilience.

### Theme 6. Social connection

Mother 11: “I stay home all day, so I barely talk to anyone … being here and chatting felt so good … it was like I’d made new friends.”

The intervention also provided a valuable social platform for pregnant women, particularly those who were homemaker or had limited social connections. This social connection helped alleviate feelings of isolation, enriched their daily lives, and renewed self-confidence through shared experiences and mutual support.

### Theme 7. Self-acceptance & self-efficacy

Mother 3:”At first I said, ‘I am not good at drawing.’ Then I figured out … once I stopped judging, I could actually do it.”

Similar to other forms of art therapy, this intervention also nurtured self-acceptance and self-efficacy. Participants learned that personal meaning mattered more than technical skill. Through painting, storytelling, and group sharing, they valued emotional expression, reduced self-doubt, and gained confidence. As they grew comfortable in the supportive setting, they embraced authentic self-expression, recognized their capabilities, and accepted their unique voices.

## Discussion

This study innovatively implemented a group-based integrative expressive art therapy intervention among pregnant women in China to improve emotional and mental health well-being. This mixed-methods study provides a comprehensive evaluation of the intervention’s effects and its value for mental health care among pregnant women with emotional difficulties in China. Quantitative and qualitative results together show significant reductions in anxiety and wide-ranging benefits, for example, emotion adjustment, self expression, enhanced peer support, and social support. The study also documents the program’s feasibility and mothers’ willingness-to-pay, which provides empirical evidence for future health-insurance reimbursement and pricing policies. In addition, the study raises public awareness of art therapy as a mental-health resource in clinical settings in China.

Mental health services stigma rather than the severity of clinical symptoms was identified as the key barrier to mental health treatment-seeking among young adults in southwestern China (13). This holds true for the sample in this study, where mental health related stigma constitutes one of the primary concerns for psychological interventions, and people with mental concerns often experience a sense of shame. Art therapy provides an valuable opportunity to initiate self-exploring, enabling the manifestation of concrete psychological concerns through creative art-making process. Furthermore, this study was conducted in an activity room within an obstetrics department, a setting that can also be considered as a “low-stigma” environment (27).

More education and campaign is needed for both mental health stigma and art therapy in China. Emerging art healing programs have recently gained popularity across regions in China, which contributed to greater public awareness for both art healing and art therapy (28), the latter of which are defined as a mental health profession, while art healing primarily targets emotional regulation rather than in-depth exploration of cognitive constructs or value frameworks. Nevertheless, the awareness of art therapy remained low in many regions cross China, especially the non-first-tier cities and regions in the western part of the country. Not surprisingly, many individuals, including the pregnant women involved in our study, perceived expressive art therapy merely as art-based activities prior to their participation. In addition to social campaigns, another important factor is physician referral. Owing to patients’ trust in their doctors, they are more likely to be recommended to participate in expressive art therapy programs; this was reflected from the feedback of participants in our study.

Plenty of previous studies have documented that both art-based interventions and art therapy can effectively alleviate anxiety and improve emotional well-being. A 2025 meta-analysis comprised 50 controlled trials demonstrated that group art intervention reduced depression and anxiety levels, with effect size comparable to conventional psychotherapy and antidepressant medications (29). In current clinical practice in China, aromatherapy, as a type of complementary and alternative therapies (CAM) is more commonly used to relieve pain and anxiety during pregnancy and childbirth (30, 31). This encourage the clinicians to recognize the benefits of CAM, as well as art therapy, which may improve patients’ overall health and satisfaction and other quality outcomes for healthcare providers. In a recent meta-analysis of 21 trials, with 7 conducted in China, Qian et al. demonstrated that art therapy interventions (10), particularly 90% of these study used music therapy, significantly alleviate perinatal anxiety and depression, with optimal outcomes achieved during labor. This study provides strong evidence that a group-based integrative expressive art therapy can be integrated into routine prenatal care, and suggest future implementations may diversify the types of art therapy/art healing programs and extend the application across both the prenatal and postpartum periods. From this perspective, our study sets an example by integrating diverse types of strategies, such as painting, storytelling, and doodling, thereby filling a gap in this area. In addition, our intervention design is grounded in a counseling framework, distinguishing it from art-based interventions. Particularly, we incorporated interpersonal therapy, narrative therapy, somatic experiencing, and mindfulness practice into the intervention sessions. These counseling technique may also contribute to the significant reductions in both state and trait anxiety. This underscores the novelty of our study and demonstrates the feasibility of delivering culturally competent, integrative expressive art therapy for mental health support among pregnant women in China. In addition, both state and trait anxiety levels declined throughout the intervention period. This five-session expressive art therapy may help participants relieve prenatal worries and develop emotion regulation skills. Since the STAI-T reflects current anxious tendencies, the substantial reduction in trait anxiety observed among the intervention group is theoretically reasonable, providing empirical evidence for the intervention effects.

Therapeutic factors and mechanisms of change in art therapies (e.g., visual art therapy, dance/movement therapy, drama therapy, music therapy) have remained understudied.

de Witte et al. summarized previous research and concluded with an overview of 19 domains of therapeutic factors (32), which included emotional elicitation and processing, symbolism &metaphor, group processes, interaction with art, connection with self, developing skills, embodiment, creativity and so on. Some of these elements were also demonstrated in both quantitative and qualitative analyses in our study. For example, one participant commented, “*While I painted, I tried to show how I felt at that moment. When I shared my pain to the group … saying it out loud helped me notice my own feelings”*, which reflected therapeutic factors in terms of group process, connection with self, and emotional elicitation. In addition to these 19 domains, we also identified cognitive behavioral change, for example as shown in **Figure 1** and **Figure 2**. Facilitated by the doodling reconstruction exercise (non-verbal communication) and sharing session (verbal communication), both foster the psychological growth of participants and enhance interpersonal communication through embodied, creative, and relational engagement (32).

Notably, participants who received expressive art therapy generally reported a more positive perception of its benefits compared with non-participants, including but not limited to improvements in emotional regulation, psychological well-being, and the quality of their relationship with their child. However, there was no significant difference in willingness-to-pay between the intervention and control groups, and both groups exhibited considerable variability in this regard. Furthermore, if this group-based expressive art therapy was covered or partially covered by health insurance, the intention to pay for both groups increased significantly. As we noticed 14.3% of pregnant women reported annual household income below 20k RMB (approximately 3k USD in 2026), which substantially limited their ability to afford medical services not covered by insurance. Particularly, in the past one year, a few cities and regions in China, such as the megacity Shenzhen, have introduced new health insurance policies to cover group-based mental health services. The findings of this real-world study provide a reference for the pricing of such medical service and for decision-making in regard to health insurance policies. Besides the favorable health policies, the shortage of qualified psychotherapists/art therapists/counselors, as well as whether certain hospitals can provide mental health services, have become another major concern during the implementation of group-based expressive art therapy in the practice.

There are several limitations in this study. First, we acknowledge this work is a preliminary study with inherent limitations due to the small sample size, low power, and single-center quasi-experimental study design, which restrict the generalizability of findings. Selection bias persists because the control and intervention groups differ in baseline anxiety and depression, and propensity score matching (PSM) cannot fully eliminate its effects on the belief about expressive art therapy and willingness-to-pay comparisons. Additionally, as this is a preliminary study, PSM serves merely as supplementary analysis. Moreover, the follow-up period for the mental health outcomes was limited, whereas the perinatal period covers a long time frame. Future studies with larger samples, multi-center recruitment, rigorous experimental designs, and longer follow-up duration are suggested to validate these findings. In addition, participants in this study reported only mild depression and anxiety at baseline, which indicates the clinical implication of the group-based integrative expressive art therapy for this target population, while those with more severe mental health issues likely require further interventions tailored to their needs, which limits the generalizability of our findings. Furthermore, due to the small sample size, we advise cautious interpretation of pricing and insurance policy conclusions from the willingness-to-pay analysis of this study.

## Conclusion

This study demonstrates the feasibility and effects of a culturally adapted integrative expressive art therapy intervention for pregnant women in China. The intervention was beneficial in reducing anxiety levels particularly, as well as enhancing emotional release, improving communication, and strengthening social support. These findings provide evidence for implementing group-based expressive art therapy among pregnant women in China, especially in health underserved regions, and offer valuable insights for policymakers regarding pricing and health insurance reimbursement for expressive art therapy services in clinical settings in China.

## Data Availability

The raw data supporting the conclusions of this article will be made available by the authors, without undue reservation.
